# Artificial Neural Network and Response Surface Methodology Modeling in Ionic Conductivity Predictions of Phthaloylchitosan-Based Gel Polymer Electrolyte

**DOI:** 10.3390/polym8020022

**Published:** 2016-01-29

**Authors:** Ahmad Danial Azzahari, Siti Nor Farhana Yusuf, Vidhya Selvanathan, Rosiyah Yahya

**Affiliations:** 1 Department of Chemistry, University of Malaya, Kuala Lumpur 50603, Malaysia; o_danny@siswa.um.edu.my (A.D.A.); farhanayusuf@siswa.um.edu.my (S.N.F.Y.); vidhya92@siswa.um.edu.my (V.S.); 2 Centre of Ionics, University of Malaya, Kuala Lumpur 50603, Malaysia

**Keywords:** phthaloylchitosan, ionic conductivity, gel polymer electrolyte, artificial neural network, response surface methodology

## Abstract

A gel polymer electrolyte system based on phthaloylchitosan was prepared. The effects of process variables, such as lithium iodide, caesium iodide, and 1-butyl-3-methylimidazolium iodide were investigated using a distance-based ternary mixture experimental design. A comparative approach was made between response surface methodology (RSM) and artificial neural network (ANN) to predict the ionic conductivity. The predictive capabilities of the two methodologies were compared in terms of coefficient of determination *R*^2^ based on the validation data set. It was shown that the developed ANN model had better predictive outcome as compared to the RSM model.

## 1. Introduction

There has been a renewed interest in chitosan as a potential polysaccharide resource owing to its specific structure and properties. Chitosan is the *N*-deacetylated derivative of chitin with a typical degree of acetylation of less than 0.35 [1]; thus, it is a copolymer of glucosamine and *N*-acetylglucosamine. There are three important polar functional groups on the chitosan polymer backbone: the hydroxyl (OH), amine (NH_2_), and ether (C–O–C), and the presence of these functional groups serves as ion exchange sites and facilitates ionic conductivity. It has been shown to have promising potential uses in devices such as in electrochemical and biosensor applications [2,3], solid state batteries, electric double layer capacitors, and fuel cells [4,5,6,7], as well as photovoltaic devices [8,9,10].

However, reasonable conductivity achieved by this material is offset by its crystallinity and its insolubility in water, and in most organic and alkali solvents. It is, however, soluble in dilute organic acids, such as acetic, formic, and lactic acids. Moreover, with long-time uses, liquid leakage of solvents from the polymer electrolyte may occur, which decreases the ionic conductivity with damage to the electrode and other components. Fortunately, the problems can be effectively circumvented by methods such as chemical modification of the chitosan. Various chemical modifications of chitosan can be made to tailor for specific applications owing to the presence of its hydroxyl and amine groups which provide reactive functional sites. Some examples are sulfonation [11], phosphorylation [12], chemical cross-linking [13], hexanoylation [14], and phthaloylation [15,16]; all of which were found to have improved features compared to the pure chitosan.

In this paper, the functionalization with phthaloyl groups to yield phthaloylchitosan (PhCh) was of interest due to its organosolubility, as we intend to use it as a component in dye-sensitized solar cell (DSSC) applications in our future work. The DSSC generally employs a solvent electrolyte and a I^−^/I_3_^−^ redox couple and have impressive energy conversion efficiencies. However, these solution-based solar cells suffer from major drawbacks such as liquid leakage. To overcome this, a lot of research has been ongoing using gel polymer electrolytes (GPE). GPEs combine the best properties of a solid and liquid electrolyte by having improved conductivities, longer life-span stabilities, and better electrode–electrolyte surface contacts. In particular, PhCh based GPE are found to be reasonably good ionic conductors. It has been shown in past studies that the choice of cation in the electrolyte plays an important role on the electrolyte/semiconductor interface electron dynamics and, hence, on the efficiency of DSSCs [9,17].

The aim of the present work is to attempt to enhance the conductivity of a PhCh based GPE using mixed iodide salt system and to study the relationship of the salt components, as well as the weight concentration that contributes towards the electrical properties of the GPE. Knowledge from this work would then be used to decide which methods to prepare the GPE for further DSSC studies based on the optimum molar ratio of the iodide mixture. Several types of salts were taken into consideration.

According to previous literature, electrolytes with iodide salt mixtures have shown better DSSC performance compared to using only a single salt system [16,17]. From these findings large sterically-hindered cations, such as tetrapropylammonium and tetrahexylammonium, led to minimized cationic conductivity but increased iodide ion conductivity in the electrolyte; cations with high charge density led to more favorable diffusion dynamics due to the small cation size. The lattice energies for the alkali metal iodides are largest for LiI (753 kJ/mol) and smallest for CsI (598 kJ/mol) [18]. It is presumable that the lattice energy of a salt could give a rough indication of its dissociability, hence its ionic conductivity, because it reflects the energy needed to separate the positive and negative ions in the salt.

**Figure 1:**
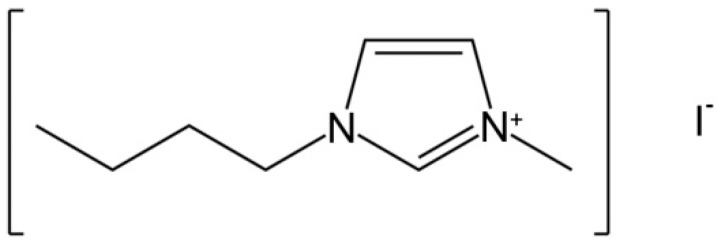
Structure of 1-butyl-3-methylimidazolium iodide.

Ionic liquids are a new class of liquids with low melting points with one such example being 1-butyl-3-methylimidazolium iodide (BMII). In spite of being a liquid, it is solely composed of ions with the bulky cation, 1-butyl-3-methylimidazolium (Figure 1). The potential of this ionic liquid for use as a nonvolatile solvent for green chemistry is of broad interest [19].

Therefore, it would be natural to explore the combined effect of using iodide salt mixtures consisting of cations of varying sizes with high charge density and low lattice energy in the electrolyte in order to benefit from all the above mentioned mechanisms. With this idea in mind, we have studied the present system by choosing BMII as the salt with a bulky cation and LiI for the salt with the smallest cation, and CsI which has the lowest lattice energy.

In general, the step-wise experimental study approach for each of the parameters involved in the preparation procedures is not only time consuming but also requires special attention in cases where there is a contribution of multiple parameters interacting simultaneously in the system. Therefore an appropriate model can be of significant interest to simulate and predict the responses from the parameters involved in the preparation process. Several approaches were used in this study to understand the behavior of the input parameters towards the output response properties of the GPE using the linear response surface methodology (RSM) and nonlinear artificial neural network (ANN). These are powerful techniques to effectively analyze the effects of several independent variables simultaneously without any knowledge on the relationship between the objective functions and variables [20,21,22]. In particular, ANN have been used to study complex phenomena, such as the electrochemical characteristics of polymer electrolyte fuel cells [20,23,24,25,26], absorption maxima of organic dyes for DSSC [27], optimization of parameter settings for the solar energy selective absorption film continuous sputtering process [28], and properties of ionic liquids [29,30]. As far as we are aware, this is the first report of a GPE employing a mixed iodide system incorporating LiI, CsI, and BMII, with the focus being the role of conductivity using RSM and ANN methods.

## 2. Materials and Methods

### 2.1. Sample Preparation

All chemicals in this work were procured from Sigma Aldrich (St. Louis, MO, USA) and used as received without further purification. The synthesis procedure for the phthaloylation of chitosan was similar to that of [31]. Chitosan (viscosity > 400 mPa·s, 1% in acetic acid at 20 °C) and excess phthalic anhydride were refluxed at 110 ± 10 °C under nitrogen atmosphere for 6 h in dimethylformamide (DMF). The reaction was allowed to proceed for another 20 h at a reduced temperature of 60 °C after which the mixture became a clear yellow viscous solution. The precipitate obtained by pouring the solution into ice-water was collected by filtration, and then further purified by redissolving in DMF and reprecipitation in ethanol. The product, phthaloyl chitosan (PhCh) was dried in vacuum at 60 °C until constant weight. Purity of the samples was validated by ^1^H NMR, δ (ppm, DMSO, 400 MHz): 7.26–8.14 (m, 4H, aromatic–H), 2.00–5.00 (m, 9H, backbone aliphatic–H).

The synthesis procedure for the ionic liquid of 1-butyl-3-methylimidazolium iodide (BMII) was done by reacting equimolar amounts of 1-methylimidazole and 1-iodobutane dissolved in cyclohexane at 70 °C under nitrogen blanket for 24 h. The resultant product was washed with *n*-hexane to remove the unreacted material. The product was dried in vacuum at 60 °C until constant weight. Purity of the samples was validated by ^1^H NMR, δ (ppm, CDCl_3_, 400 MHz): 9.89 (s, 1H, –NC**H**=N^+^–), 7.52 (dd, 2H, –N–C**H**=C**H**–N^+^–), 4.32 (t, 2H, –C**H_2_**–N–), 4.10 (s, 3H, =N–C**H_3_**), 1.90 (m, 2H, –CH_2_–C**H_2_**–CH_2_–), 1.37 (m, 2H, –CH_2_–C**H_2_**–CH_3_), 0.95 (t, 3H, –CH_2_–CH_2_–C**H_3_**).

The gel polymer electrolyte (GPE) was prepared by stirring well 0.4 g of ethylene carbonate (EC), 0.4 g of DMF, and *w* g of salt system where *w* is the sum of predetermined amounts of lithium iodide (LiI), caesium iodide (CsI), and BMII. Subsequently, 0.1 g of PhCh was added into the salt solution. This mixture was vigorously stirred and heated to 60 °C and the procedure was halted when it became a homogeneous gel. After cooling to room temperature, iodine (10% of *w*) was added and stirred until a homogeneous GPE was obtained.

**Figure 2:**
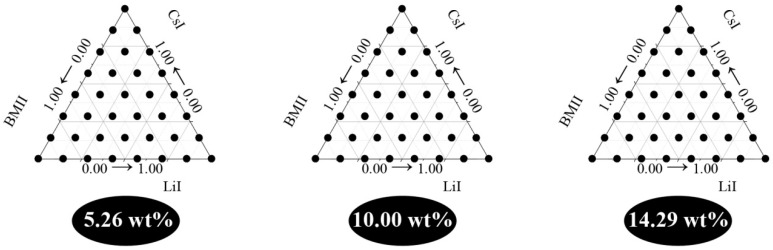
Design of experiment for the preparation of the PhCh–(LiI:CsI:BMII) GPE.

Preliminary runs for the GPE preparation were done with *w* values in increments of 0.05 g from 0.00 g (0.00 wt %) to 0.40 g (30.77 wt %) using a salt system of LiI:CsI:BMII in a ratio of 0.33:0.33:0.33. However, it was found that the system was only miscible up to 0.15 g (14.29 wt %) salt content. Therefore, to analyze the effects of the independent variables simultaneously, the design of experiment (DoE) employed consisted of process variables (in this work, three levels were used corresponding to 5.26 wt %, 10.00 wt %, and 14.29 wt %) and mixture variables. As shown in Figure 2, for each level, 36 samples were prepared. Samples located on the vertex of the ternary diagram represent a single component system, those on the edges are binary mixtures and any sample points inside the ternary diagram are ternary mixtures). The sequence of experimental preparation and measurement runs were randomized to minimize bias in the readings obtained.

### 2.2. General Characterization

^1^H NMR measurements were performed with an ECA 400 MHz spectrometer (JEOL, Tokyo, Japan). Attenuated Total Reflectance-Fourier Transform Infrared (ATR-FTIR) spectra were recorded with a Spotlight 400 spectrometer (PerkinElmer, Beaconsfield, Bucks, UK). The acquisition parameters were done with a total of 128 accumulations at 2 cm^−1^ resolution with a spectral range from 4000–600 cm^−1^.

### 2.3. Electrochemical Impedance Spectroscopy (EIS)

**Figure 3:**
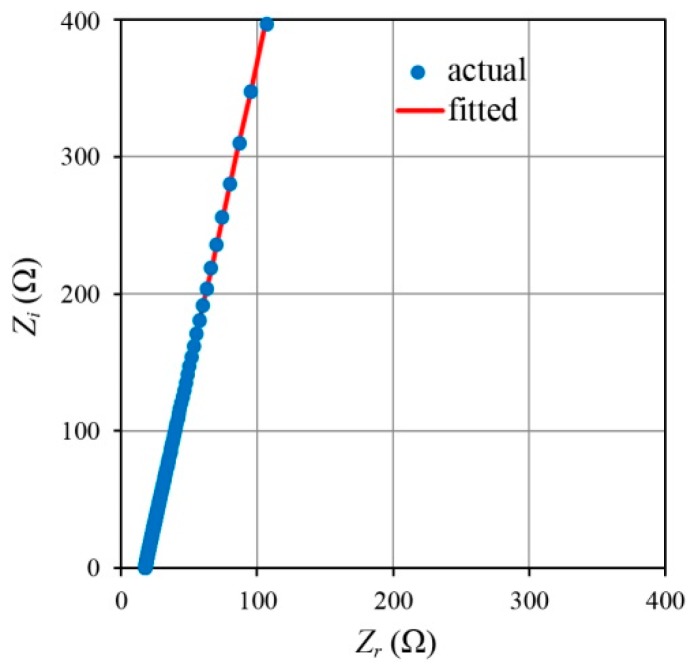
Typical Nyquist plot for the PhCh–(LiI:CsI:BMII) GPE.

Electrochemical impedance spectroscopy (EIS) measurements were done using an IM3590 instrument (HIOKI, Nagano, Japan) from 50 Hz to 200 kHz with an average of three replicate readings at each experimental point. A sample cell holder with two stainless steel disc electrodes 1 cm in diameter was used to sandwich the GPE with a thickness of 0.13 cm. The impedance data was processed in a complex impedance plot where the imaginary part *Z*_i_ was plotted against its real part *Z*_r_. Nyquist plots generated by impedance measurements can normally consist of (i) a depressed semicircle; (ii) a tilted spike or (iii) a depressed semicircle with a tilted spike. All the results in this work however, showed a Nyquist plot behavior with only a tilted spike as shown in Figure 3.

The Nyquist plot was fitted (using a non-linear least squares method using Microsoft Excel’s Generalized Reduced Gradient solver algorithm) to an equivalent circuit consisting of a resistor and a constant phase element (CPE) in series to obtain *R_B_*, the bulk impedance at the intersection of the plot with the real impedance axis. The real and negative imaginary parts of the Nyquist plot consisting of a tilted spike are given by the equations [32]:

(1) Zr=RB+cos(πp2)Qωp

and

(2) Zi=sin(πp2)Qωp

where ω is the angular frequency, *Q* is the capacitance due to the electrical double layer (EDL) formed at the electrode/electrolyte interface during the impedance measurement [33] and *p* which has a value between 0 and 1 is the skew parameter that controls the degree of inclination of the tilted spike from the *Z*_r_ axis. Hence, the ionic conductivity of the samples was calculated using the following equation:

(3) $$\sigma =\frac{2d}{R_{B}A}$$

where *d* is the half thickness of the sample, and *A* is the electrode/electrolyte contact area.

Apart from conductivity (σ) values, other PhCh–(LiI:CsI:BMII) GPE electrical properties were also evaluated. Bandara and Mellander [32] have shown that, using only impedance measurements, it is possible to obtain the number density and mobility of charge carriers since in principle, the conductivity is the product of these parameters. Arof *et al.* [34] have further developed and derived in detail a method based on this to determine the diffusion coefficient (*D*), ion mobility (μ) and number density of the charge carriers (*n*). Briefly, the diffusion coefficient (*D*) was given as:

(4) $$D=\frac{{\left(Q^{-1}\varepsilon_{r}\varepsilon_{0}A\right)}^{2}}{\tau_{2}}$$

where ε_r_ is the dielectric constant of the electrolyte, ε_0_ is the vacuum permittivity (8.85 × 10^−14^ F·cm^−1^) and $\tau_{2}$ is $1/\omega_{2}$ with $\omega_{2}$ being the angular frequency corresponding to the minimum in the imaginary impedance, *Z_i_*. From the impedance data, ε*_r_* for each sample can be obtained by a plot of the real part of the complex permittivity, ε*_r_*, *versus* frequency, *f*. The impedance data was substituted in the equation below:

(5) $$\varepsilon_{r}=\frac{Z_{i}}{\left({Z_{r}}^{2}+{Z_{i}}^{2}\right)}\left(\frac{d}{\omega \varepsilon_{0}A}\right)$$

It was observed that all the samples showed a constant value between log *f* = 4.5 and log *f* = 5.0. Hence, the value of the dielectric constant, ε_*r*_, for all the samples was taken at the high frequency of 200 kHz. The mobility (µ) of the charge carriers can then be determined from the Nernst–Einstein relation:

(6) $$\mu =\frac{eD}{k_{b}T}$$

where $k_{b}$ is the Boltzmann constant (1.38 × 10^−23^ J·K^−1^), *T* is the absolute temperature in Kelvin and e is the electron charge (1.602 × 10^−19^ C). Finally, the number density of charge carriers (*n*) can be obtained using the following equation:

(7) $$n=\frac{\sigma }{\mu e}$$

## 3. Results and Discussion

### 3.1. FTIR Spectra of the PhCh–(LiI:CsI:BMII)

**Figure 4:**
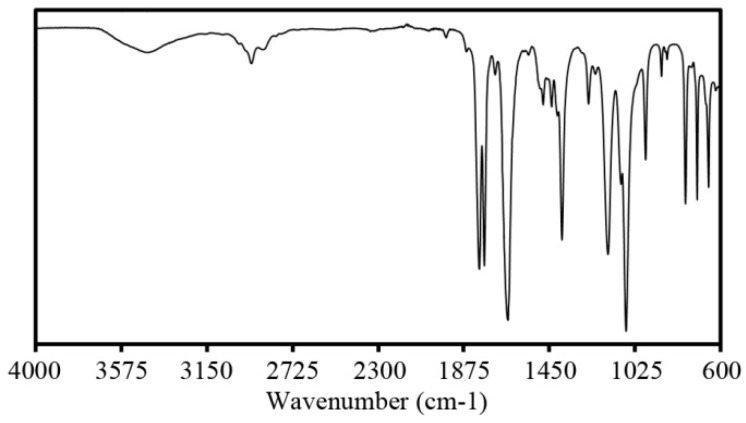
FTIR spectra of the PhCh–(LiI:CsI:BMII) GPE.

**Figure 5:**
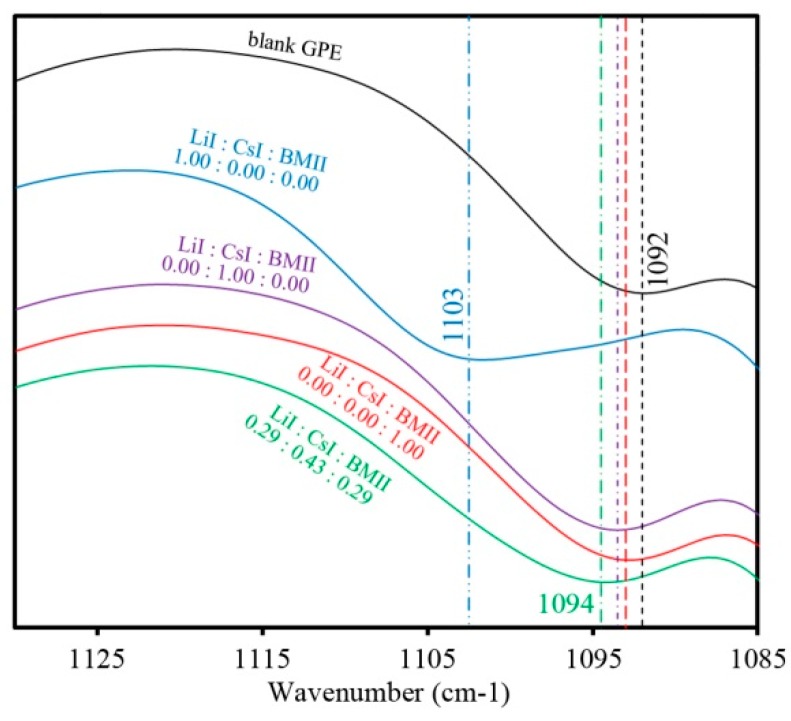
FTIR spectra of selected PhCh–(LiI:CsI:BMII) GPE samples for the ether absorption bands ranging from 1085 to 1130 cm^−1^.

**Figure 6:**
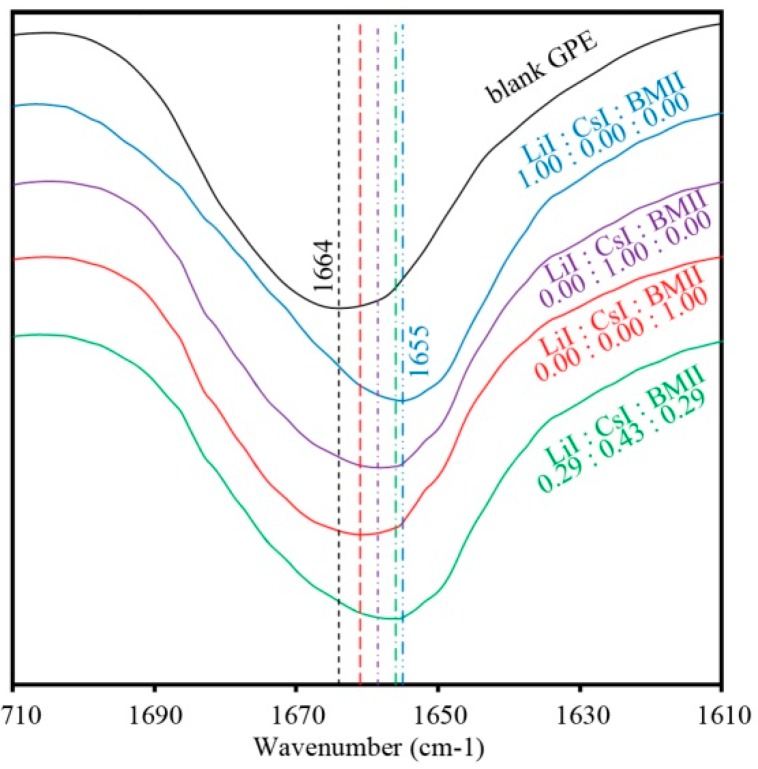
FTIR spectra of selected PhCh–(LiI:CsI:BMII) GPE samples for the amide absorption bands ranging from 1610 to 1710 cm^−1^.

Figure 4 shows the FTIR spectra that is typical of the PhCh–(LiI:CsI:BMII) GPE samples. Peaks attributed to the ether C–O–C group appeared in the range of 1000 to 1120 cm^−1^. Samples with different salt contents showed a peak that varied in position from 1092 to 1103 cm^−1^ (Figure 5) implying that there is a coordination of salt ions to the polymer matrix. The peak at 1256 cm^−1^ is due to C–N asymmetric stretching mode. The intense peak around 1660 cm^−1^ is attributed to the amide group presence in the GPE. This peak also tends to shift to lower wavenumbers when varying salt contents are added to the system as seen in Figure 6 further confirming coordination interactions of the salts in the polymer [35]. The strong peak at 1773 and 1798 cm^−1^ is due to carbonyl C=O stretching. The peak at 2930 cm^−1^ is due to the CH_3_ symmetric stretching mode. The broad peak in the range of 3200 to 3700 cm^−1^ corresponds to the O–H and N–H group of the GPE.

### 3.2. Surface Modeling

The effects of the four different input variables on the responses of conductivity (σ), diffusion coefficient (*D*), and number density of charge carriers (*n*) were studied. The first, second, and third variable is the mixture ratio of LiI, CsI, and BMII, respectively, in the GPE system. The fourth variable is the sum wt % of the salt system. Table 1 shows the complete experimental design of the factor combinations, together with the experimental response outputs. Experimentally, the highest conductivity was obtained for the sample with LiI:CsI:BMII in ratio of 0.00:0.71:0.29 at 14.29 wt % with σ = 1.495 × 10^−2^ S·cm^−1^.

**Table 1:** DoE for the independent variables used in this study along with the observed response.

			σ (×10^−2^ S·cm^−1^)			*D* (×10^−6^ cm^2^·s^−1^)			*n *(×10^18^ cm^−3^)		
LiI ratio	CsI ratio	BMII ratio	5.26 wt %	10.00 wt %	14.29 wt %	5.26 wt %	10.00 wt %	14.29 wt %	5.26 wt %	10.00 wt %	14.29 wt %
0.43	0.57	0.00	0.913	1.313	1.332	34.83	180.40	185.24	42.25	11.71	11.57
0.43	0.43	0.14	0.898	1.249	1.374	14.79	118.45	348.48	98.06	17.04	6.35
0.43	0.29	0.29	0.906	1.314	1.439	37.90	142.42	306.05	38.59	14.87	7.59
0.57	0.43	0.00	0.912	1.307	1.326	49.96	301.30	285.20	29.38	6.98	7.58
0.57	0.29	0.14	0.917	1.290	1.386	25.43	204.88	302.36	58.01	10.14	7.79
0.57	0.14	0.29	0.967	1.278	1.371	50.76	297.61	200.65	30.73	6.91	11.06
0.71	0.29	0.00	0.938	1.295	1.348	39.03	168.79	181.47	38.66	12.71	12.36
0.71	0.14	0.14	0.940	1.288	1.338	24.12	214.20	233.37	62.75	9.68	9.28
0.71	0.00	0.29	0.935	1.348	1.295	21.09	246.71	221.55	71.65	8.85	9.65
0.86	0.14	0.00	0.987	1.289	1.307	59.82	242.16	194.78	26.55	8.58	10.95
0.86	0.00	0.14	0.972	1.278	1.289	35.05	228.26	169.68	44.69	9.01	12.62
1.00	0.00	0.00	0.985	1.264	1.270	48.09	198.48	222.75	33.17	10.25	9.18
0.29	0.29	0.43	0.883	1.298	1.415	23.23	214.89	365.06	61.49	9.72	6.29
0.29	0.43	0.29	0.831	1.317	1.424	16.27	190.98	370.30	82.13	11.13	6.48
0.14	0.43	0.43	0.874	1.296	1.448	17.37	93.58	265.67	80.93	22.30	9.10
0.14	0.57	0.29	0.851	1.298	1.452	34.80	88.54	382.44	39.37	23.84	6.33
0.00	0.57	0.43	0.825	1.277	1.455	20.20	99.26	140.14	65.73	20.71	17.21
0.29	0.57	0.14	0.912	1.295	1.403	45.18	168.66	266.22	32.56	12.39	8.92
0.29	0.71	0.00	0.879	1.339	1.380	15.35	153.54	219.23	92.24	14.06	10.33
0.00	0.71	0.29	0.794	1.292	1.495	7.76	71.91	356.08	164.91	28.93	6.80
0.14	0.71	0.14	0.835	1.297	1.445	14.07	110.76	155.18	95.60	18.87	15.03
0.14	0.86	0.00	0.859	1.312	1.424	13.31	150.49	241.17	103.96	14.03	9.76
0.00	0.86	0.14	0.847	1.277	1.466	29.36	66.24	256.99	47.80	31.12	9.22
0.00	1.00	0.00	0.806	1.280	1.433	10.67	95.69	118.54	121.46	21.53	19.71
0.43	0.14	0.43	0.889	1.276	1.363	53.79	82.04	156.36	26.60	25.09	14.36
0.57	0.00	0.43	0.933	1.314	1.248	31.63	197.06	113.00	47.51	10.99	17.78
0.00	0.43	0.57	0.836	1.269	1.406	13.63	50.09	136.36	98.67	40.87	16.61
0.14	0.29	0.57	0.828	1.326	1.364	14.27	114.07	146.85	93.46	18.73	15.76
0.29	0.14	0.57	0.879	1.267	1.351	21.73	199.37	159.29	65.23	10.00	14.06
0.43	0.00	0.57	0.926	1.293	1.240	37.34	159.30	155.07	39.96	13.06	12.89
0.00	0.29	0.71	0.759	1.222	1.345	4.51	32.77	141.64	270.96	60.05	15.80
0.14	0.14	0.71	0.831	1.258	1.336	14.12	79.94	180.58	94.68	25.34	11.90
0.29	0.00	0.71	0.823	1.239	1.234	15.92	100.72	125.95	83.32	19.82	15.78
0.00	0.14	0.86	0.779	1.188	1.318	11.88	48.46	128.09	105.62	39.85	16.57
0.14	0.00	0.86	0.815	1.229	1.314	8.89	96.66	131.55	148.58	20.53	16.49
0.00	0.00	1.00	0.785	1.213	1.281	7.64	66.66	148.05	165.32	29.31	14.12

The analysis, evaluation and estimation of the accuracy and applicability for the polynomial models were determined with Design Expert^®^ Software Version 6.0.6. In the data preprocessing step, all the input variables in Table 1 (for $x_{1}$, $x_{2}$, $\;x_{3}$, and $x_{4}$ representing LiI, CsI, BMII ratios and the sum wt % of the salt system, respectively) were normalized into dimensionless values according to the equation below:

(8) $$X_{\text{new}}=a+\frac{\left(X_{\text{old}}-X_{\text{min}}\right)\left(b-a\right)}{\left(X_{\text{max}}-X_{\text{min}}\right)}$$

where $x_{i}\in \left(a;b\right)$

$X_{\text{new}}$ is the normalized input variable;
a is the new minimum value of the normalized input variable;
  b is the new maximum value of the normalized input variable;
$X_{\text{old}}$ is the original input variable;
$X_{\text{min}}$ is the minimum value of the original input variable;
$X_{\text{max}}$ is the maximum value of the original input variable.

For the RSM procedure in this work, a=0 and  b=1 for the mixture variables and a=−1 and  b=1 for the process variable. The response outputs can then be evaluated with the different types of response surface models (*i.e.*, linear, two-factor interaction (2FI), quadratic, reduced cubic, and full cubic) to compare the appropriateness of each model. The response surface models consist of polynomials with coefficients and would take the general form as follows:

(9) $$y\left(x,w\right)=w_{0}+\overset{D}{\underset{i=1}{\sum }}w_{i}x_{i}+\overset{D}{\underset{i=1}{\sum }}\overset{D}{\underset{j=1}{\sum }}w_{ij}x_{i}x_{j}+\overset{D}{\underset{i=1}{\sum }}\overset{D}{\underset{j=1}{\sum }}\overset{D}{\underset{k=1}{\sum }}w_{ijk}x_{i}x_{j}x_{k}+\ldots$$

(not all of the coefficients are independent due to interchange symmetries amongst the *x* variables). In practice, to capture complex dependencies in the data, a higher-order polynomial may need to be used. The coefficient of determination *R*^2^ indicates how well the data fits a statistical model where a good fit will have values close to 1. The use of an adjusted *R*^2^ takes into account the phenomenon of the *R*^2^ spuriously increasing when additional terms are added to the RSM model and its value is ≤*R*^2^. Unlike *R*^2^, the adjusted *R*^2^ increases when a new term is added only if the new term improves the *R*^2^ more than would be expected by chance.

The analysis of variance (ANOVA) done on each of the response data revealed that there were model terms with Prob > *F* values that were greater than 0.1 indicating that their contribution to the model was not significant. To improve the model fit, a backward regression procedure was performed from the full cubic model. The unnecessary terms which had high Prob > *F* values were removed stepwise. From this procedure, it was found that all the RSM for conductivity (σ), diffusion coefficient (*D*), and number density of charge carriers (*n*) followed a crossed reduced cubic × quadratic model (corresponding to the crossed DoE for mixture variable × process variable). The ANOVA also revealed that in the case of *D* and *n* which had a high max to min response ratio, a power transform to the data set was required to stabilize the variance and make the data more normal distribution-like. A Box-Cox plot was used to determine the suitable power transform and it was recommended as follows [36]:

(10) $$y^{\prime }={\text{log}}_{10}y$$

where *y’* is the transformed response and *y* is the original response.

Table 2 lists the outcomes for the optimized models for each of the responses. The signal to noise (S/N) ratio compares the range of the predicted values at the design points to the average prediction error and typically, ratios greater than 4 indicates adequate model discrimination [37]. In this case, all the S/N ratio values are well above 4; therefore, the design space can be navigated by the selected model. These models can be used to predict the response values within the limits of the experiment. The final equation for the developed models in Table 2 in terms of coded (normalized) factors is as follows:

*σ* (×10^−2^ S·cm^−1^) = 1.267 *x*_1_ + 1.256 *x*_2_ + 1.180 *x*_3_ + 0.144 *x*_1_*x*_2_ + 0.276 *x*_1_*x*_3_ + 0.157 *x*_1_*x*_4_ + 0.162 *x*_2_*x*_3_ + 0.315 *x*_2_*x*_4_ + 0.251 *x*_3_*x*_4_ − 0.129 *x*_1_*x*_4_^2^ − 0.120 *x*_2_*x*_4_^2^ − 0.154 *x*_3_*x*_4_^2^ − 0.449 *x*_1_*x*_2_*x*_3_ − 0.089 *x*_1_*x*_2_*x*_4_ − 0.113 *x*_1_*x*_3_*x*_4_ + 0.096 *x*_2_*x*_3_*x*_4_ − 0.198 *x*_1_*x*_2_(*x*_1_-*x*_2_) + 0.125 *x*_2_*x*_3_(*x*_2_-*x*_3_) − 0.180 *x*_1_*x*_2_*x*_4_^2^ − 0.249 *x*_1_*x*_3_*x*_4_^2^ − 0.009 *x*_2_*x*_3_*x*_4_^2^ + 1.034 *x*_1_*x*_2_*x*_3_*x*_4_ + 1.482 *x*_1_*x*_2_*x*_3_*x*_4_^2^ + 0.287 *x*_1_*x*_2_*x*_4_^2^(*x*_1_-*x*_2_) (11)

log_10_ [*D* (×10^−6^ cm^2^·s^−1^)] = −3.648 *x*_1_ − 4.085 *x*_2_ − 4.161 *x*_3_ + 0.535 *x*_1_*x*_2_ + 0.370 *x*_1_*x*_3_ + 0.294 *x*_1_*x*_4_ − 0.620 *x*_2_*x*_3_ + 0.583 *x*_2_*x*_4_ + 0.547 *x*_3_*x*_4_ − 0.403 *x*_1_*x*_4_^2^ − 0.300 *x*_2_*x*_4_^2^ − 0.349 *x*_3_*x*_4_^2^ + 2.933 *x*_1_*x*_2_*x*_3_ + 0.153 *x*_2_*x*_3_*x*_4_ + 1.094 *x*_2_*x*_3_(*x*_2_-*x*_3_) + 1.136 *x*_2_*x*_3_*x*_4_^2^ (12)

log_10_ [*n* (×10^18^ cm^−3^)] = 18.961 *x*_1_ + 19.396 *x*_2_ + 19.447 *x*_3_ − 0.518 *x*_1_*x*_2_ − 0.325 *x*_1_*x*_3_ − 0.236 *x*_1_*x*_4_ + 0.628 *x*_2_*x*_3_ − 0.461 *x*_2_*x*_4_ − 0.444 *x*_3_*x*_4_ + 0.351 *x*_1_*x*_4_^2^ + 0.232 *x*_2_*x*_4_^2^ + 0.267 *x*_3_*x*_4_^2^ − 2.722 *x*_1_*x*_2_*x*_3_ − 0.079 *x*_2_*x*_3_*x*_4_ − 1.045 *x*_2_*x*_3_(*x*_2_-*x*_3_) − 1.059 *x*_2_*x*_3_*x*_4_^2^ (13)

**Table 2:** Optimized models for each of the response properties.

Response	RSM *R*^2^	RSM Adjusted *R*^2^	S/N Ratio	4-*h*-1 ANN *R*^2^
Conductivity, *σ*(×10^−2^ S·cm^−1^)	0.9919	0.9897	65.14	0.9936
log_10_ Diffusion coefficient, log_10_ [*D* (×10^−6^ cm^2^·s^−1^)]	0.9218	0.9091	28.07	0.9317
log_10_ number density of charge carriers, log_10_ [*n* (×10^18^ cm^−3^)]	0.8920	0.8743	23.91	0.8989

**Figure 7:**
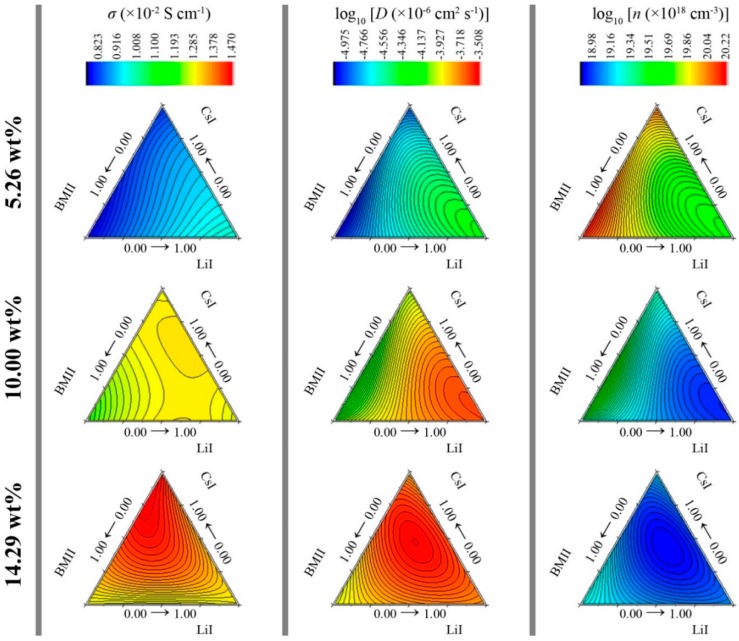
Plot of predicted RSM for σ (×10^−2^ S·cm^−1^), log_10_ [*D* (×10^−6^ cm^2^·s^−1^)], and log_10_ [*n* (×10^18^ cm^−3^)] at various salt concentration levels.

The RSM models are graphically represented in Figure 7. It can be seen that as the sum wt % of the salt system increases from 5.26 to 14.29 wt %, the conductivity increases. The position for the mixture ratio combination of LiI, CsI, and BMII that led to the highest conductivity varies for each level. At 5.26 wt %, the σ was highest when LiI ratio was the highest. As the sum wt % of the salt system increased further, the highest σ for each level shifted along the ridge of the LiI-CsI region and at 14.29 wt %, the ratio of LiI:CsI:BMII was 0.01:0.81:0.18 showed highest σ at 1.467 × 10^−2^ S·cm^−1^. Since the conductivity is the product of the ionic mobility and number density, it can be inferred from Figure 7 that the contribution towards conductivity was mainly due to the high diffusion of the free ions in the GPE rather than the number density of the charge carriers. The *n* values decrease as the concentration of the salt system increases.

**Figure 8:**
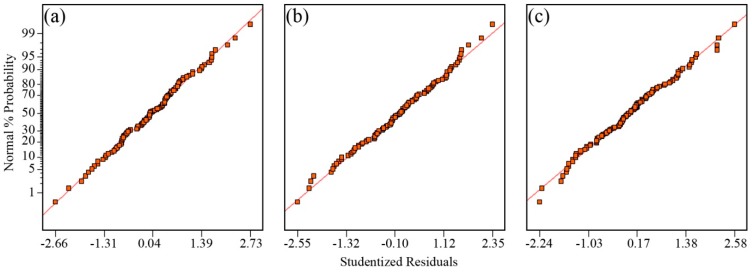
Normal probability plot of residuals for (**a**) σ (×10^−2^ S·cm^−1^); (**b**) log_10_ [*D* (×10^−6^ cm^2^·s^−1^)]; and (**c**) log_10_ [*n* (×10^18^ cm^−3^)].

**Figure 9:**
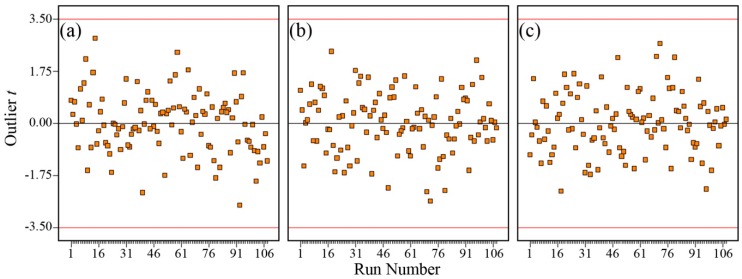
Outlier *t* plot for (**a**) σ (×10^−2^ S·cm^−1^); (**b**) log_10_ [*D* (×10^−6^ cm^2^·s^−1^)]; and (**c**) log_10_ [*n* (×10^18^ cm^−3^)].

The normal probability plots in Figure 8 show that the residuals generally fall on a straight line implying that the errors are distributed normally for σ, log_10_ *D*, and log_10_ *n*. Figure 9 shows that the scatter plot across the graphs does not exceed the threshold of the outlier *t*. These graphical diagnostic checks imply that the models that have been developed do not violate the independence or constant variance assumption.

An ANN-based model was also developed during the present study as an alternative to the polynomial RSM, which provides the modeling of complex nonlinear relationships for describing the response for σ, log_10_ *D*, and log_10_ *n*. ANN, inspired by the structural and/or functional aspect of a biological neural network, has attracted increasing attention in recent years, particularly for process modeling [20,23,24,25,26]. It consists of an input layer (independent variables), a number of hidden layers and an output layer (dependent variables). Each of these layers consists of a number of inter-connected processing units called neurons. These neurons are linked to one another along weighted connections which are passed into the hidden layer. The hidden layer then does all the data processing and produces an output based on the sum of the weighted values from the input layer modified by a sigmoid transfer function. In this study, a hyperbolic tangent-sigmoid transfer function was applied:

(14) $$f\left(x\right)=-1+\frac{2}{\left(1+e^{-2x}\right)}$$

**Figure 10:**
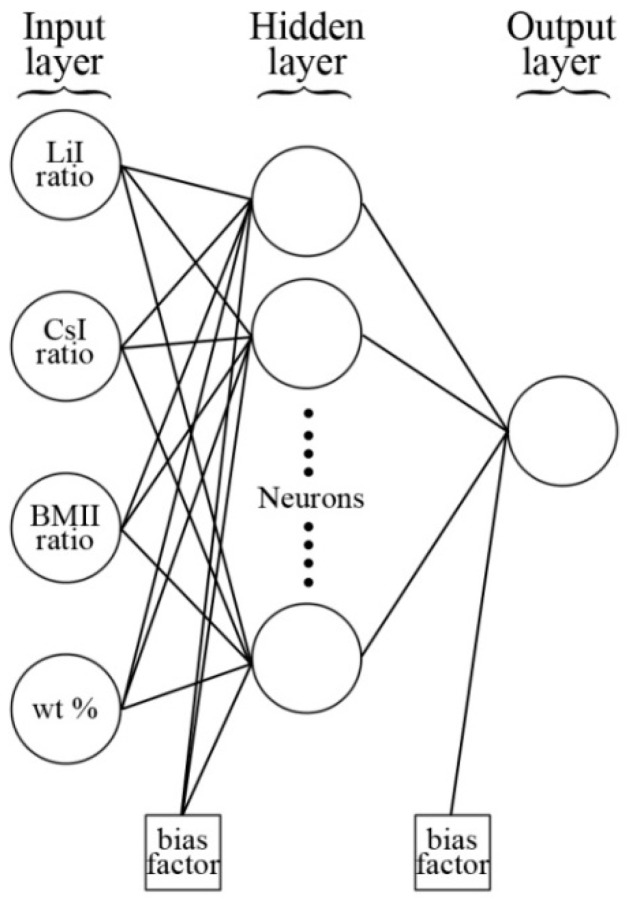
General scheme of the artificial neural network.

The Levenberg–Marquardt back-propagation algorithm was used for network training. The same input and output factors that was used in the RSM approach was fed into the ANN model. In order to achieve fast convergence to the minimal mean square error (MSE), the inputs and outputs were scaled within the uniform range of *a* = −1 to *b* = 1 by following Equation (1) to ensure uniform attention during the training process. The Neural Network Toolbox of MATLAB Version 7.12 (R2011a) was used in all ANN calculations. The general scheme of the neural network is shown in Figure 10.

Designing the topology of the network became the first step for training the neural network. For simplicity, each network was developed to represent only one type of response (from Table 1) at a time. Therefore, the topology of the ANN developed was designated as 4-*h*-1 *i.e.*, four input neurons representing the four variables for the preparation of the PhCh–(LiI:CsI:BMII) GPE, *h* represents the number of hidden neurons in a single hidden layer, and one output neuron representing one type of response. Using the experimental data, the ANN topologies were built, trained, tested, and validated with a number of hidden layers varying from 1 to 9. The training process was run by a trial and error search method until an optimum model was found and the minimum of mean square error was reached in the validation process. 

**Figure 11:**
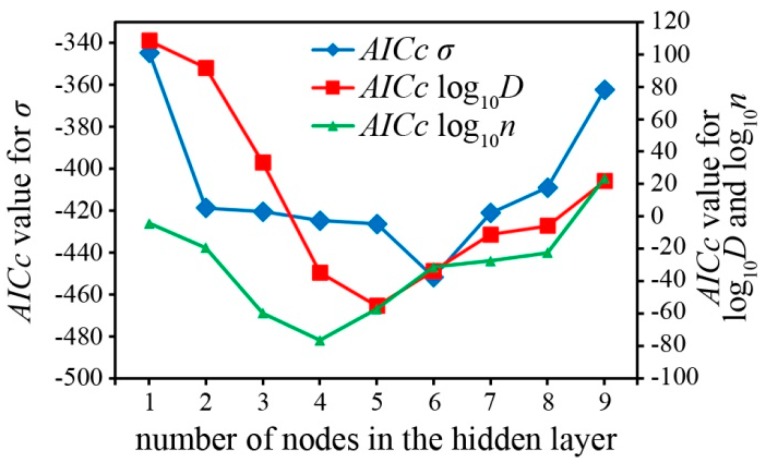
*AICc* for all the response outputs.

**Figure 12:**
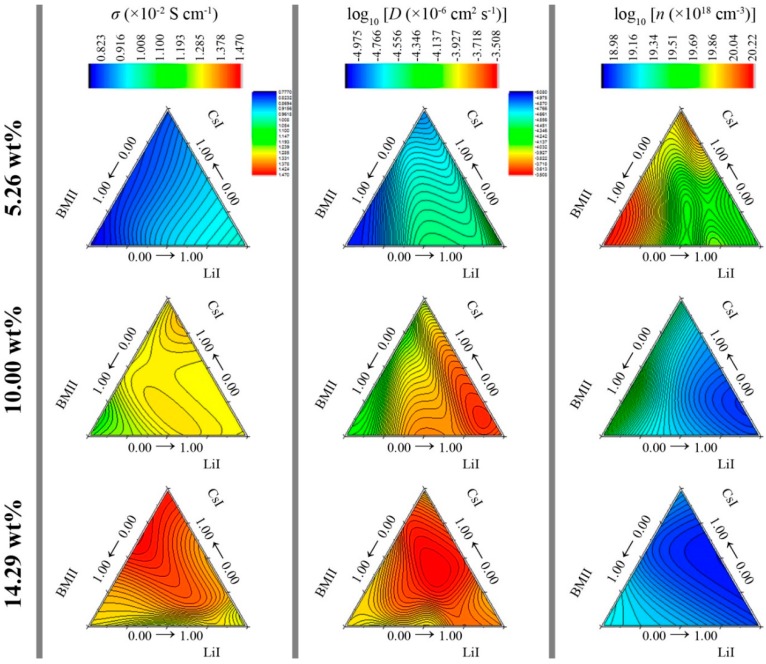
Plot of predicted ANN for σ (×10^−2^ S·cm^−1^), log_10_ [*D* (×10^−6^ cm^2^·s^−1^)], and log_10_ [*n* (×10^18^ cm^−3^)] at various salt concentration levels.

Similar to the case of developing RSM models, over-fitting of the model could occur when an excessive number of hidden neurons are used. Furthermore, it has been pointed out previously [38,39] that the conventional use of the *R*^2^ or adjusted *R*^2^ is not a sufficient measure for the goodness of fit in nonlinear models. To supplement this deficiency, we used the Akaike Information Criterion (AIC) [40,41,42], a model selection method that is widely accepted for measuring the validity within a cohort of nonlinear models [43]:

(15) AIC=2p−2ln(L)

where *p* = number of parameters and ln(*L*) = maximum log-likelihood of the estimated model. The latter, in the case of a nonlinear fit with normally distributed errors, is calculated by:

(16) $$\text{ln}\left(L\right)=0.5\times \left(-N\times \left(\text{ln}2\pi +1-\text{ln}N+\text{ln}\overset{n}{\underset{i=1}{\sum }}{r_{i}}^{2}\right)\right)$$

with *r*_1_, ..., *r*_n_ = the residuals from the nonlinear least-squares fit and *N* = their number. For small sample sizes, the bias-corrected *AIC* variant is given as (*AICc*):

(17) $$AICc=AIC+\frac{2p\left(p+1\right)}{n_{\text{size}}-p-1}$$

where *n*_size_ is the sample size. Operationally, the best model is selected from the smallest *AICc* value computed for each of the 4-*h*-1 models. The calculated *AICc* values for the ANN networks of the response outputs are shown in Figure 11, and in all cases it was observed that the number of hidden nodes that would be sufficient to build the topology of the neural network without over-fitting for the models of σ, log_10_ *D*, and log_10_ *n* were six, five, and four, respectively. The ANN models are graphically represented in Figure 12. Although the contour shapes of the ANN appears to be comparable to the RSM, the *R*^2^ calculated from the developed 4-*h*-1 ANN models as shown in Table 2, indicate that the the 4-*h*-1 ANN models have a better fit over the RSM models. The ANN model showed highest σ at 1.467 × 10^−2^ S·cm^−1^ from a LiI:CsI:BMII ratio of 0.01:0.63:0.36 at 14.29 wt %.

### 3.3. Determination of the Importance of Each Input Variable

Once the response models have been developed, it would be of interest to determine which of the four input variables (mixture ratio of LiI, CsI, BMII, and the sum wt % of the salt system) would influence the properties of the prepared PhCh–(LiI:CsI:BMII) GPE the most. The fit of the RSM models are limited by the fact that it is constrained to have a polynomial shape, whereas the ANN surface model has the advantage that it can accommodate non-linear shapes. Typically, the importance of each input variable in RSM models can be evaluated quite simply by the weight coefficients where positive values indicate that the corresponding input would increase the value of the response output and vice versa. The *F*-value can also be used where the larger its magnitude for the corresponding coefficient terms, the more significant is the contribution of the corresponding coefficient term [44]. However, the weight coefficients in the ANN models cannot be used directly in a similar manner with the RSM methods. Many researchers that adopted the ANN approach have also labeled it as a “black box” because they provide little explanatory insight into the relative influence of the independent variables in the prediction process [45,46,47,48]. There should still be some other way to extract information about the relative importance that the input variables have on the output since the ANN can be regarded as analogous to real biological neurons, where each weight influences the proportion of the incoming signal that will be transmitted into the neuron’s body. 

One method adopted for the determination of relative contribution of input factors was by using the partial derivatives (PaD) [49]. The sensitivity of network outputs according to small input perturbations is represented by the Jacobian matrix d*y*/d*x*^t^ = [∂*y*/∂*x*]_m×n,_ which links the modification of inputs, *x_j_*, and the variation of outputs, *y_j_ = f*(*x_j_*) . For a network with *n* inputs, one hidden layer with *n_i_* nodes, and one output (*i.e.*, *m* = 1), the gradient vector of *y_j_* with respect to *x_j_* when a hyperbolic tangent sigmoid function is used for the activation is *d_j_* = [*d_j1_*,…,*d_je_*,…,*d_jn_* ]*^T^*, with:

(18) $$d_{\text{je}}=s_{j}\overset{n_{h}}{\underset{h=1}{\sum }}w_{eh}w_{ho}\left(1-{I_{hj}}^{2}\right)$$

where *s_j_* = (1 − *f*(*x*)^2^) is the derivative of the output node with respect to its input, *I_hj_* is the output of the *h*th hidden node for the input *x_j_*, the scalars *w*_e*h*_ and *w_h_*_o_ are the weights between the *e*th input node and the *h*th hidden node, and between the *h*th hidden node and the output node. The partial derivatives can then be interpretable, but it has to be evaluated at a large, representative sample of points from the input space. Due to the small experimental dataset (36 samples × 3 levels), a larger dataset consisting of 4851 points (231 samples × 21 levels) was randomly generated within the input space to obtain information representative of the developed models. The next step was to summarize this large collection of numbers to a single measure of importance for each input to report an average value. This was done by the sum of the square partial derivatives obtained per input variable:

(19) $$SSD_{i}=\overset{N}{\underset{j=1}{\sum }}\left({d_{ji}}^{2}\right)$$

**Figure 13:**
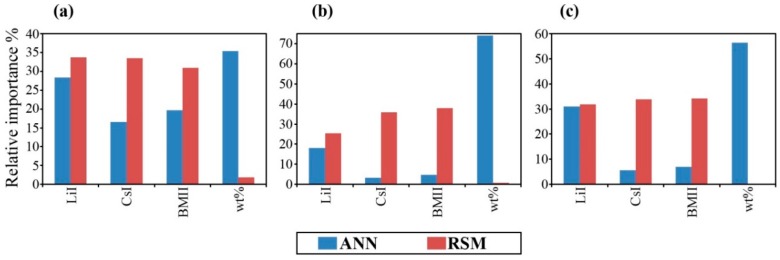
PaD relative importance% of single input factors towards the output response properties of (**a**) σ (×10^−2^ S·cm^−1^); (**b**) log_10_ [*D* (×10^−6^ cm^2^·s^−1^)]; and (**c**) log_10_ [*n* (×10^18^ cm^−3^)].

The calculated relative importance of the input variables is shown in Table 3 and Figure 13. A similar treatment was adopted for the RSM models using PaD for comparison. RSM PaD generally showed little sensitivity for mixture variables whereas ANN PaD showed distinctly that LiI had the highest contribution and although CsI and BMII contribution was similar, CsI had the lowest contribution. This is possibly due to Li^+^ being the smallest cation, which would have greater mobility in the system. The ANN PaD showed reasonably that the process variable for the sum wt % of the salt system had significant contribution to the response properties compared to the RSM PaD which showed negligible effect.

**Table 3:** PaD relative importance of individual input towards the output response properties of (a) σ (×10^−2^ S·cm^−1^); (b) log_10_ [*D* (×10^−6^ cm^2^·s^−1^)]; (c) log_10_ [*n* (×10^18^ cm^−3^)] in terms of percentages.

Variable	σ (×10^−2^ S·cm^−1^)		log_10_ [*D* (×10^−6^ cm^2^·s^−1^)]		log_10_ [*n* (×10^18^ cm^−3^)]	
	ANN PaD%	RSM PaD%	ANN PaD%	RSM PaD%	ANN PaD%	RSM PaD%
*x*_1_, LiI	28.40	33.73	18.04	25.35	31.01	31.83
*x*_2_, CsI	16.52	33.50	3.22	35.88	5.57	33.91
*x*_3_, BMII	19.68	30.91	4.81	37.94	7.04	34.24
*x*_4_, wt %	35.39	1.85	73.94	0.84	56.38	0.02

Furthermore, to analyze the contribution of all possible two-way interactions of input variables a similar method to the modification (PaD2) proposed by [50] was used:

(20) $$d_{j12}=\left(1-f{\left(x\right)}^{2}\right)\left[\left(-2f\left(x\right)\right)\overset{n_{h}}{\underset{h=1}{\sum }}w_{1h}w_{ho}\left(1-I_{hj}{}^{2}\right)\overset{n_{h}}{\underset{h=1}{\sum }}w_{2h}w_{ho}\left(1-{I_{hj}}^{2}\right)\;+\overset{n_{h}}{\underset{h=1}{\sum }}w_{1h}w_{2h}w_{ho}\left(-2I_{hj}\right)\left(1-I_{hj}{}^{2}\right)\right]$$

where *w*_1*h*_ and *w*_2*h*_ are, respectively, the weights between the first studied input neuron and the *h*th hidden neuron, and between the second studied neuron and the *h*th hidden neuron.

**Figure 14:**
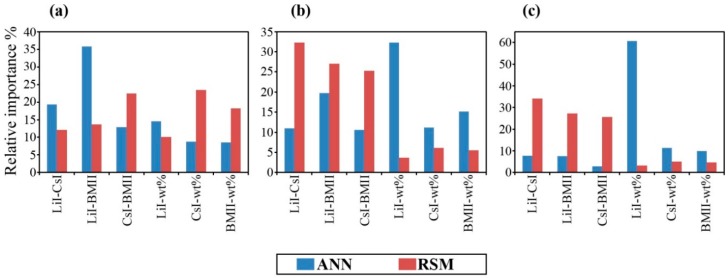
PaD2 relative importance% of two-way interactions towards the output response properties of (**a**) σ (×10^−2^ S·cm^−1^); (**b**) log_10_ [*D* (×10^−6^ cm^2^·s^−1^)]; (**c**) log_10_ [*n* (×10^18^ cm^−3^)].

Although the shape of the contour fit in the RSM was comparable to the ANN model, it appears that the ANN has a slightly different approach for the interpretation of the contribution of two way interactions compared to the RSM (Table 4 and Figure 14). This result is not unexpected since the ANN model is not hindered by the polynomial constraints of the RSM model. The better fit of the ANN gives a hint on the salt-salt compatibility where LiI-BMII was the highest contributor compared to CsI-BMII in RSM. The ANN PaD2 also showed that the individual salt concentration contribution was highest with LiI but CsI for RSM. It would seem that the RSM PaD and PaD2 tends to favor CsI contribution since the electrical properties in the fitted ternary contours showed it was highest along the ridge of the LiI–CsI region. On the other hand, ANN PaD and PaD2 showed that the contribution was favored from LiI even though the ternary contours showed the best properties near the CsI region (at the highest salt concentration of 14.29 wt %). This should not be construed as counterintuitive however, since as described earlier in the preliminary runs for the GPE preparation, it was observed that the salt system was no longer miscible at salt concentrations above 14.29 wt %. Although the LiI-CsI-BMII salt system in the GPE would behave synergistically to give the best conductivity near the CsI region, this is only true at the concentration level of 14.29 wt %. It is possible that at much higher concentrations, the compatibility of CsI with LiI and BMII could hinder its solubility in the salt system. Thus, it has been shown the better predictive power that ANN has over the RSM approach.

**Table 4:** PaD2 relative importance of two-way interactions towards the output response properties of (a) σ (×10^−2^ S·cm^−1^), (b) log_10_ [*D* (×10^−6^ cm^2^·s^−1^)], and (c) log_10_ [*n* (×10^18^ cm^−3^)] in terms of percentages.

Variable	σ(×10^−2^ S·cm^−1^)		log_10_ [*D* (×10^−6^ cm^2^·s^−1^)]		log_10_ [*n* (×10^18^ cm^−3^)]	
	ANN PaD2%	RSM PaD2%	ANN PaD2%	RSM PaD2%	ANN PaD2%	RSM PaD2%
*x*_1_*x*_2_, LiI-CsI	19.30	12.08	11.01	32.34	7.75	34.18
*x*_1_*x*_3_, LiI-BMII	35.78	13.71	19.78	27.04	7.49	27.28
*x*_2_*x*_3_, CsI-BMII	12.92	22.44	10.63	25.33	2.88	25.73
*x*_1_*x*_4_, LiI-wt %	14.60	10.15	32.25	3.71	60.62	3.23
*x*_2_*x*_4_, CsI-wt %	8.82	23.44	11.20	6.08	11.28	4.97
*x*_3_*x*_4_, BMII-wt %	8.58	18.18	15.14	5.50	9.98	4.61

## 4. Conclusions 

The preparation of a gel polymer electrolyte system based on phthaloylchitosan was successfully prepared using lithium iodide, caesium iodide, and 1-butyl-3-methylimidazolium iodide as the mixed salt system. From the EIS analyses, it was found that the range of conductivities obtained experimentally was from 0.759 × 10^−2^ to 1.495 × 10^−2^ S·cm^−1^. The ANN model showed better predictive capabilities compared to the RSM approach. The highest conductivities were obtained in the ternary region near CsI at 14.29 wt %. However, relationship studies of the contributing input factors showed that LiI has the highest compability in the mixed salt system.

## Supplementary Materials

The following are available online at www.mdpi.com/2073-4360/8/2/22/s1. Figure S1. (a) Appearance typical of the GPE prior to adding iodine. Note that the gel is capable of holding the magnetic stirring bit in place without falling to the bottom; and (b) appearance typical of the GPE after adding iodine. Note that the gel appears to be covering the entire length of the vial because the vial was tilted horizontally during stirring after being stirred vertically standing to ensure a more thorough homogeneous mixing; Figure S2. The complete EIS measurement setup using a HIOKI IM3590 instrument.
