# Supplementary material for: Artificial Neural Network and Response Surface Methodology Modeling in Ionic Conductivity Predictions of Phthaloylchitosan-Based Gel Polymer Electrolyte
Source: Polymers (Basel). 2016 Jan 29;8(2):22. doi: 10.3390/polym8020022 (PMC6432590; doi:10.3390/polym8020022)
Supplement: Supplementary file 1 [file polymers-08-00022-s001.pdf]

Polymers **2016**, *8*, 22

Supplementary Materials: Artificial Neural Network and Response Surface Methodology Modeling in Ionic Conductivity Predictions of Phthaloylchitosan Based Gel Polymer Electrolyte

Ahmad Danial Azzahari, Siti Nor Farhana Yusuf, Vidhya Selvanathan and Rosiyah Yahya

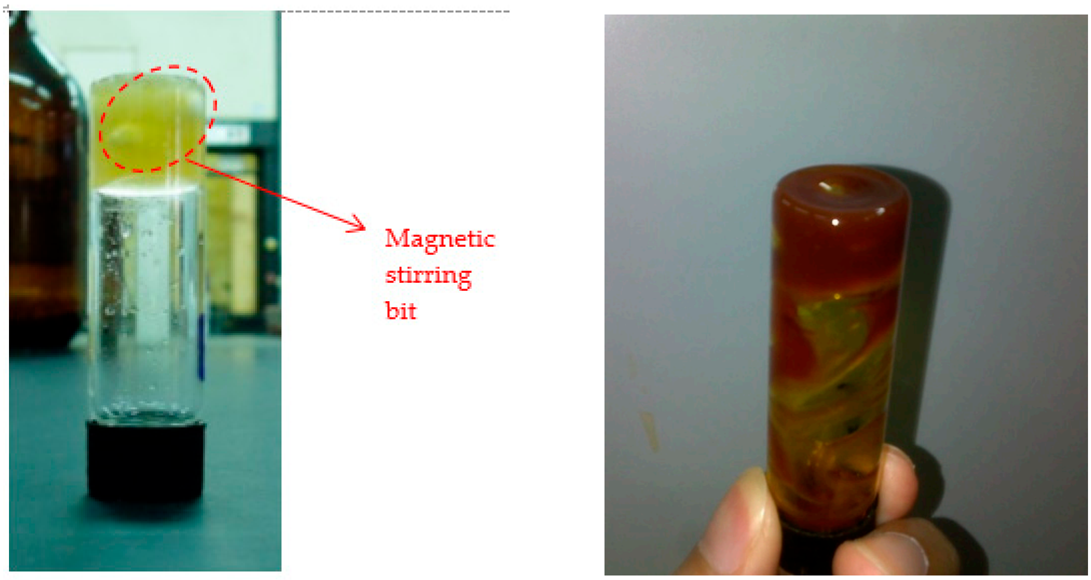

(**a**) (**b**)

Figure S1. (**a**) Appearance typical of the GPE prior to adding iodine. Note that the gel is capable of holding the magnetic stirring bit in place without falling to the bottom; and (**b**) appearance typical of the GPE after adding iodine. Note that the gel appears to be covering the entire length of the vial because the vial was tilted horizontally during stirring after being stirred vertically standing to ensure a more thorough homogeneous mixing.

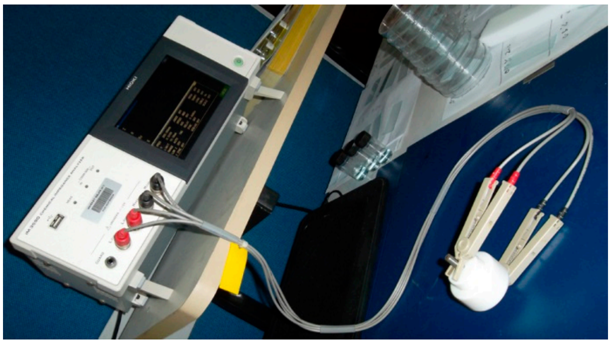

Figure S2. The complete EIS measurement setup using a HIOKI IM3590 instrument.

© 2016 by the authors; licensee MDPI, Basel, Switzerland. This article is an open access article distributed under the terms and conditions of the Creative Commons by Attribution (CC-BY) license (http://creativecommons.org/licenses/by/4.0/).
